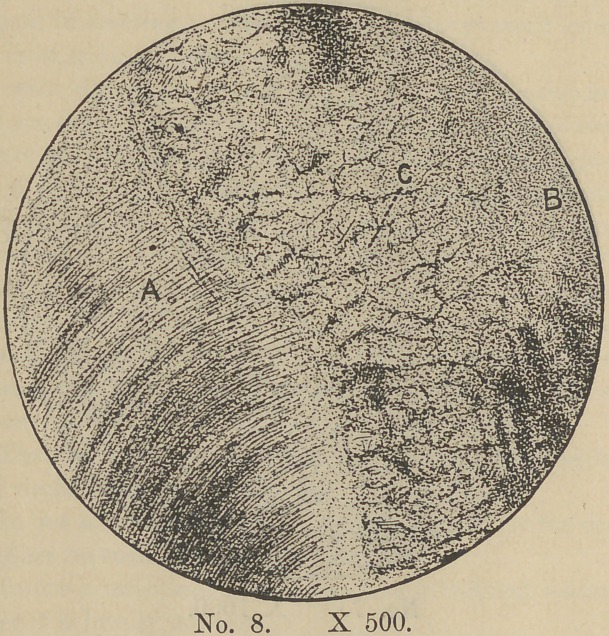# Dentogony

**Published:** 1888-06

**Authors:** W. C. Brittan

**Affiliations:** Detroit


// THE DENTAL REGISTER.

VOL. XLII.] JUNE, 1888. [No. 6.

### COMMUNICATIONS.

### Dentogeny.

BY DR. W. C. BRITTAN, DETROIT.

Read before the Section on Dental and Oral Surgery, American Medical Association, May, 10th, 1888.

The first indication of tooth development in the human embryo is noticeable at a period somewhere about the fortieth day of foetal life, and consists simply of an increased thickening of the epithelium over that part which is to form the future alveolar border.

At this time there may be found, sometimes, not always, upon the lingual surface a slight depression or groove, the "dentinal groove" of writers which, when present, consists of an infolding only of the mucous tissue, and is usually more marked anteriorally than elsewhere. As however it is not always present, any histological importance can not reasonably be attached to it. Later a change is seen to occur at certain points in the cells of the epithelium, consisting of an enlargement of the cells and their nuclei, these points corresponding nearly to the position of the future teeth. This changed epithelial structure dips down into the adjacent embryonic tissue in the form of "cords" or rather of tubular glands (Plate 1). The cells of the outer layer of these cords have a columnar form, yet they are seen to have now lost much of their resemblance to those of the columnar layer of the epethelium with which they are said to be identical. Plate II. shows one of these cords highly magnified.

These epithelial cords constitute the embryonic enamel elements and later form what is known as the "enamel organ" which is concerned mainly—but not wholly—in the production of the

No. 1. X 75.

enamel of the tooth. Coincidently with the above changes an increased activity has been in progress in the submucous tissue adjacent to these cords, resulting in a multiplication of its cells, and a consequent increased density of this tissue at points which occur sometimes beneath the cords, sometimes at one side (Plate 1—d) and often one upon each side, in which case the enamel elements for two seperate teeth of the same type are furnished from one cord.

No. 2. X 300.

These cell clusters of the submucous tissue gradually assume a papilliform appearance, pushing up against the overlying cord in which they soon become nearly enveloped and now constitute the so-called "dentine germ, or organ." In these two germs—the enamel and the dentine—we have the prime factors of tooth-building. And the processes here employed furnish problems, many of which are yet unsolved. The interpretations which we shall give are solely the result of our own study of these tissues. The

No. 3. X 50.

micrographic illustrations are from some of the preparations used for that purpose, and the preparations themselves are from the human embryo. If we err in any of the statements here made, it is quite consoling to know that all other writers upon this subject have done the same. We believe however that we are well fortified.

Rapid developmental changes now occur. The cord, by a separation of its walls, assumes a somewhat "stirrip-like" form, its base conforming to the contour of the "dentine organ" until by the upward growth of the same, all that part which is to compose the crown of the tooth is covered by the enamel organ in the form of a cap (Plate III). which now separates itself from the original cord by the breaking up of that structure.

In this connection we would suggest that the prevailing dogma that the enamel organ for the second set of teeth is supplied from the cords of the first is incorrect. As a matter of fact they are derived directly from the mucous epithelial layer, just as in the first instance.

All the above changes occur previous to deposit of either enamel or dentine, but we now have both these organs in a state of development nearly sufficient to begin that work, and from this point the progress of development is much slower than previously.

We will now by referring to Plate III. note some of the changes that have taken place in the "enamel organ" in its transition from the cord with its outer wall of columnar cells to its present condition. By the separation of its walls in the course of its development the sells of its interior have by growth and separation and attachment to one another by their processes, now assumed the form of stellate cells, and now constitute that part of the organ known as the "stellate reticulum" (b) the exact function of which is not understood. This structure occupies all that part lying between its inner and outer investments, viz: the wall in contact with the dentine organ (d) and that in contact with the saculus proper (e) which latter is the product of the contiguous embryonic tissue. Further that at the point where the enamel organ embraces the cervical portion of the embryonic tooth (g) the wall of the organ is folded upon itself, thus bringing both walls (d and e) into mutual contact. These walls, although originally one and the same, here diverge and now form two distinct and well differentiated structures. We also note that the outer investment (e) has lost all appearance of a columnar cell layer which characterized the original cord, and is now composed of a system of vessels and capillaries distributed through a

No. 4. X 300.

fibrous membrane whose sells (mostly fusiform) now lie parellel to its surface.

At the inner wall (d) a very great change has also taken place.

Here the original columnar form of the cells is still preserved at this stage of development, although greatly reduced in size and having also lost other marked features of structure. Plate IV. gives a very good illustration of this boundary highly magnified.

The changes above mentioned as occuring in these two investments, might with some show of reason be accounted for as due somewhat to mechanical influences. For instance, the outer wall (e—Plate III) in assuming its convex form in which we now find it, would, so to speak, be put upon the stretch. In consequence its cells would be forced into a horizontal position. On the other hand there would be a crowding together of the cells in assuming a concave form in the inner wall (d) thus maintaining their columnar form. These walls (d and e) or boundaries are said to be identical with the so-called "Malpighian" layer of the epithelium, the columnar cells of which (d) are here transformed into "ameloblasts," or enamel-forming cells.

To ascertain, if possible, how far this is a fact, let us examine a little into some of the structural characteristics of the tissues here involved. The saculus, as before intimated, is the product of the surrounding embryonic tissue and exhibits two well differentiated structures, the outer layer being composed of fibrous connective tissue elements of a somewhat loose texture, while the inner, or that in contact with the outer investment of the reticulum, is in structure much finer, and contains a profusion of nucleated bipolar cells with long and highly refractive processes, and lying mainly paralled to the surface of the organ in the outer wall of which they seem to be interwoven, and to connect upon the other side directly with the processes of the stellate cells of the reticulum. Again the processes of these cells form a like connection with those of the "stratum intermedium," and these last are seen to form true axial connections with the columnar cells of the ameloblastic layer, or, as we prefer to call it, the enamel matrix (Plat III d). At the point of juncture of the cells of the stratum intermedium with those of the matrix, they are joined by lateral processes. Sometimes two or more of these joinings are seen, thus forming those transverse lines, which mark the upper boundary of the matrix cells, which are again joined at their lower ends where they come into contact with the dentine organ in just the same manner; and thus is formed the transverse line which marks the boundary here. And thus also is formed the

No. 5. X 1000.

supposed membrane lying between the enamel and the dentine, and which consists simply of a mesh or network composed of the lateral processes of the matrix cells. These matrix cells also send axial processes beyond this border line and into the dentine organ, where nucleated enlargements occur in them. Thus they become those columnar bodies known as "odontoblasts" to which we shall refer again furthur on.

We know that in them as in the case of all the other tissues of the body, whatever elements are required for the work of building are derived from blood constituents. We know also that the enamel organ is extra vascular, the nearest approach of blood capillaries being at its outer investment (Plate III—e) on the one side, and the dentine organ on the other. Therefore it is clear that such supply must come from the point first mentioned. The blood constituents passing through the reticulum, probably there receive such elaboration as fit them for their final place of deposit. Herein then consists the function of this stellate structure.

An examination of the section represented in Plate III, shows that a change is in progress in the nuclei of the stellate cells in the immediate vicinity where a deposit of enamel is about to occur—noticeable as a marked increase in size. They now appear as highly refractive spherical bodies of an opalescent character. Coincidently with or a little before, the first deposit of enamel two or more courses of these bodies make their appearance in the upper part of the matrix cells (Plate V—d) where they have been termed the nourishing cells of the enamel organ. These spherical cells, or nuclei, are not however developed within the enamel matrix, but are identical with those of the reticulum (c) and probably constitute the calcific elements of the enamel. They, by some means, find way within the matrix cells where, superimposed one upon another, by some unknown law, coalescence occurs, thus forming the rods or prisms of the enamel, the matrix cell walls becoming the cement substance between the same. In this we have a perfect explanation of the striated appearance of the enamel rods—an appearance which cannot be reasonably accounted for in any other way. It is a well known fact that by treating the enamel with alkalies, the rods may be devided into minute cubical sections just in the places where these striations occur. We submit therefore that each of these little sections represents an original spherical body or nucleus which was developed within a stellate cell of the reticulum.

Associated with the first deposit of the enamel which occurs nearly coincidently with that of the dentine another very marked change takes place, consisting of an increased density in that part of the reticulum lying in contact with the matrix cells (Plate V—b). This occurs in consequence of the upward push of the growing tooth-germ, accompanied with the increasing enamel depo-

No. 6. X 75.

sit which now so encroaches upon the reticulum that it here becomes folded or gathered back upon itself, thus forming that line known as the stratum intermedium (Plate V—e). In Plate IV it will be noticed this structure is not yet developed. In consequence of this folding back of this structure it may be presumed that the nuclei lying in the deeper parts of the organ are brought into more intimate association with the matrix cells thus favoring their migration. Plate V gives quite a good illustration of this. Here a portion of the matrix (d), stratum intermedium (b), and and the reticulum (c), have been torn out from the enamel organ and left attached to the already formed dentine (a). As a result also of the outward push of the growing tooth, the matrix associated with what remains of the reticulum and its outer investment becomes an attenuated layer over the apex of the tooth, and gradually assumes that form over the whole crown, as the process of deposit is completed. In this we have the mythical membrane of Nasmyth.

At this stage the enamelization of the tooth is nearly completed, but the function of the organ or rather what now remains of it does not end here, as we shall see. In plate VI we have represented an embryonic deciduous canine tooth with its investments at about the above stage of development. In making this section the enamel organ and the layer of last formed enamel was torn from its position, making quite a space (c) upon the labial side. Upon the palatine side, the enamel organ (b) is intact but with a disarrangement of the last formed enamel (e); yet quite a thick layer still covers the dentine, distinguishable from the latter by its lighter color (f). At the base of the rudiment is seen a dark knotted line (h) composed of vessels and capillaries in cross section. Here is the growing point of the tooth. It is at this point that the crown begins and at this point that the root is finished. Immediately below this line is the base of the saculus, and in contact with that the forming jaw-bone (i). We note that the line (h) is joined by the lower borders of the enamel organ (b) thus completing the circuit. At this point of juncture (k) the two walls of the organ are in mutual contact. A little higher up at (j) is the curvical line of the tooth where the enamel is to end. Yet we see that the organ extends quite a distance below this point, in fact nearly surrounds the dentine germ. An examination of the section reveals the fact that the nuclei in the reticulum are not developed in that structure below the points (j). Therefore this fact taken in connection with the general conformation of the parts clearly indicate that at this point (j) the tooth crown is to be finished and all below that is the developing root. And it is just as evident also that in its outward growth the tooth passes directly through the remains of what once was the enamel organ, and which now becomes the matrix for the cement substance of the root and the boundary line for the dentine of the same. Inasmuch as this deposit here begins much earlier than that of the cement it is seen that such a boundary must in some way be provided.

Here probably ends the office of the enamel organ so far as developmental function is concerned. Its life's activities having been spent in erecting to past usefulness a monument composed of the most enduring tissue of the whole body.

The main disinguishing features between the dentine and the enamel are chemical and structural. The same chemical elements enter into the composition of both, but in different proportions. This fact alone would account for a variation in structure, yet aside from this there are other important reasons for this. As we have seen, a specific organ is employed in the production of each, each working in harmony with the other, and both for the consummation of the final result. In view of this, one would be led to suspect a similarity in their modes of working. In studying these structures the following differences of structure are most prominent:

1. That while one is interiorally absolutely non-vascular the other is decidedly vascular.

2. In one a matrix is provided as a receptacle for the deposit; in the other no such provision is made.

3. In one a reticulated structure is a prominent feature; in the other it is hardly noticeable.

4. In one the deposit occurs in a distinct and specific form; in the other it is semi-homogeneous.

Notwithstanding however these well marked differences which we believe have often heretofore led investigation wide of the truth we are convinced that there is very little, if any difference in the mode of working in each. The dentine organ, as before stated, is developed in, and probably from, the embryonic elements of the submucous tissue. Sometime previous to its envelopment by the enamel organ a rapid development of blood vessels occurs at its base. These shoot upward into its substance rapidly becoming a dense and arborescent system. They run mainly parallel to its long axis with numerous fine branchings as they approach its upper boundary where they appear to end in loops. The substance of the organ appears as a granular mass—due to its somewhat dense cellular structure. Its cells are small, variously formed, and are joined by very delicate processes, thus forming another stellate structure analagous to that of the en-

No. 7. X 1000.

amel organ and probably also with the same functional characteristics, although with a widely differing environment. Sometime previous to the deposit of dentine there occurs what has been supposed to be a metamorphosis of the cells along the border line in contact with the enamel matrix which now assumes the appearance of a columnar layer at this point. These bodies, the so-called "odontoblasts" as stated above, are only nucleated enlargements of the axial processes of the matrix cells of the enamel organ which penetrate the dentine organ along this border and are again by fine processes joined to the deeper cells of that structure, thus forming a continuous system. The axial connections of these columnar bodies with the matrix cells form the "dentinal sheaths" or tubuli—their axis cylinders—the dental fibres. The branchings of the "tubuli" are a result of a fusion of two or more of these bodies. We are aware that all this is very unorthodox, but so we see it. In this connection we would ask a careful study of Plate VII. This section was made from a growing tooth. The space (a) was occupied by the den-

No. 8. X 500.

tine pulp. The "odontoblasts", so-called (b), are left attached by their long processes to the formed dentine. As represented here their bodies often penetrate deeply into the dentine organ. As a rule however they lie nearly in contact with the line of deposit. The delicate processes at the free ends of these bodies by which they connect with the structural elements of the dentine pulp are torn away.

An examination of the dentine pulp at the time when deposit is in progress reveals the fact of a profusion of granular bodies or nuclei, quite similar to those of the enamel organ which here as there we may infer constitute the calcific elements of the deposit. The reticulum of the dentine pulp is by no means as easy of demonstration as that of the enamel. Very careful manipulation and fresh unmounted specimens are the conditions of success in this direction. Plate VIII represents such a structure in the pulp of an adult tooth. Since however this occurs where there had been a secondary deposit an abnormal growth is to be suspected.

Upon examining the pulp of a fresh specimen of growing tooth we find adjacent to the inner extremities of the odontoblasts a profusion of the granular nuclei referred to above as constituting the elements of deposit. In just what manner this deposit is brought about is unknown; but the facts that it occurs in layers or laminae, and also that these granular constituents do not lose their identity in the new formation are easy of demonstration. By treating the dentine with alkalies we may separate it into its component parts in just the same order in which they were put together. This we have demonstrated before.

The chemical difference existing between the enamel and dentine seems more easy of a reasonable explanation. In the dentine organ exist just those conditions requisite to cause this difference. With its dense system of blood vessels enclosed in unyielding walls are furnished the conditions for excessive blood pressure required for the passage of those elements (coloids) by transudation or otherwise, which make this difference.

Since writing the above it has, through some subsequent observations, occurred to me—almost to the point of conviction—that in its inceptive development the dentine germ is indebted wholly to the enamel organ for the stimuli requisite for such a purpose. In other words the enamel organ is the inceptor of the dentine germ.

We know that no tooth is developed without the pre-development of such an organ. Even in that type of teeth which have no enamel, the formation of an analagous enamel organ is the first inception. This is a fact which can not be explained by natural or other selection, or by referring to primitive forms. Its existence therefore, under such circumstances, would seem to imply that of necessity it must bear some significant connection with the formation of the dentine germ.

DISCUSSION ON THE ABOVE PAPER WAS OPENED BY
DR. M. H. FLETCHER, M.D., D.D.S.

*Mr. President and Gentlemen:*

The paper we have just heard and the accompanying illustrations evidently represent the expenditure of much labor and time in their preparation. It is only those who are initiated and experienced in such work that can appreciate the hours of research; thought and reasoning necessary in such an investigation. In the whole subject of embryology, and the various phenomena connected with it, one will not find more natural barriers and difficulties to overcome in any branch than in undertaking the study of the dental system of animals. It has claimed much attention from our most learned and talented scientists, and is still involved in great obscurity. The most excellent paper just presented clearly indicates a love for the work which would accomplish great achievements in this direction under favorable circumstances and opportunities.

The illustrations shown in connection with the essay represent various stages of development from the dipping down of the mucus membrane, to the development of both enamel and dentine. As to the time of this first appearance and dipping down the essayist does not differ from other investigators. But the "dental groove" which he speaks of as not always being present should be called the "dental ridge." Sudduth says on this point: "Concomitant with the formation of the ridge the proliferation of the cells of the infant layer causes a depression of the sub-epithelial layer lying immediately underneath. Were we to lift up this thickened epithelial layer it would leave behind a groove in the under-lying tissue. But let it be remembered that in lifting up the ridge or rampart of epithelial cells we have made the groove. It is never a groove per se, but, when formed, is always an artificial product which can be made at will." The "dental groove of writers," as quoted, is therefore now obsolete.

The essayist also says that at this period "A change is seen to occur at certain points in the cells of the epithelium consisting of an enlargement of the cells and their nuclei, these points corresponding nearly to the position of the future teeth." This enlargement of the cells and nuclei at this stage has not to my knowledge been noted by other writers. But some authors speak of a proliferation of the cells, their size remaining the same until the dipping down of the cord has occurred, at which time some investigators have claimed also that the cells of the Malipighian layer assume a columnar form. However, the latest investigations seem to show the cells of these layers to be oval or cylindrical shaped until the cord comes into contact with the dentine papillæ, and, after this occurs, they take on the columnar form and evidently become the ameloblasts.

The stirrup shape of the enamel organ spoken of is only the form seen on making sections of the tissue. As a matter of fact the organ is bell-shaped forming a hood over the dentine papilla. It is spoken of as a cap however in another part of the paper. At this stage the Doctor shows a section in which he claims that, "At the point where the enamel organ embraces the cervical portion of the embryonic tooth the wall of this organ is folded upon itself thus bringing both walls into mutual contact." (See Fig. 3.) So far as I know no other author has observed this. Other investigators represent this portion as free from such conditions as described. But since this is not a matter closely concerned in any part of the development it is not of great significance.

The important point in the paper and that which has the greatest claim for originality, is the theory of the origin of the odontoblasts. After speaking of the numerous lateral processes given off by the ameloblasts or "matrix cells," he says: "And thus is formed the supposed membrane lying between the enamel and the dentine and which consists simply of a mesh or network composed of the lateral processes of the matrix cells. These matrix cells also send axial processes beyond this border line, and into the dentine organ where nucleated enlargements occur in them. Thus they become those columnar bodies known as odontoblasts." This certainly gives to the odontoblasts an entirely new origin, for they have up to the present time been considered as a modification of the cells of the sub-epithelial tissue in like manner as the ameloblasts are a modification of the

Malipighian layer. The layer of cells at the summit of the dentine papilla which come into nearest contact with the enamel organ are the ones thus converted into odontoblasts.

To claim that any lateral process of a cell "becomes nucleated" and forms a cell of another class is certainly a perversion of the laws of morphology to some extent; for it would seem that if there are any truths established in this branch of science it is that, when nucleated cells divide, the division of the nucleus—*as a rule at least*—precedes that of the whole cell. If the Doctor could show that the matrix cells generate a second nucleus which passes through this basement membrane then it would add greatly to the rationality of his theory. The development of the cell however is a gradual process from a general to a special state. We can conceive how the sub-epithelial layer, in the process of differentation, might develop from the epithelium, or the reverse. But the processes of amelification and dentification are, according to the essayist's own definition quite different, and, in consequence, would require different kinds of cells—at least sufficient difference to need the step from epithelial to sub-epithelial structure before these cells could be converted into organs differing as widely as the enamel organ and the dentine organ. Then, too, in this particular case, there is a great chance of error, for these processes are extremely hard to trace. Legros and Magitot say in regard to the process of the stellate cells: "It is a remarkable fact that no line of juncture can be discovered where these cells are connected with each other, the various reagents failing to disclose the least trace of it." And this certainly holds good with regard to any process given out by the ameloblasts at this stage. Nevertheless this new theory does credit to its author and probably may be true. But the chances are against it.

The fact that the enamel organ is to be universally found in the earliest stages of tooth development, even in that type of teeth which have no enamel, is not proof of the truth of this theory. For it is certain that many cells act catalytically on their environments. It is therefore not improbable that this action is the influence given out from the enamel organ and is necessary for the incipient development of the odontoblast layer.

Reverting to the subject of the enamel organ, we may say that the description is good of the manner in which enamel is probably formed by the "super-imposing one upon another of the spherical cells or nuclei from the 'stratum intermedium' within the walls of the matrix cells." And also that the lateral processes of the cells and the cell walls may form the boundaries to the enamel prisms.

One illustration (No. 6—B) shows the enamel organ with its stellate reticulum to be present over the particular part of the tooth in which the enamel is being deposited. But Sudduth claims there is no deposit of enamel until the recticulum disappears. The illustration, if properly understood, would disprove Sudduth's theory on this point. That the remains of the enamel organ form Nasmyth's membrane seems to be pretty well established. But the essayist seems to look upon the existence of this membrane as a myth.

The Doctor would make this enamel organ a most versatile affair. For according to his theory it first prepares and forms the enamel, then gives the necessary cells to the dentine organ. Without it he thinks dentine could not be produced. And before it ends its existence he has it enclosing the root of the tooth, and producing the cement which is much less like enamel than dentine. This latter function of the enamel organ is certainly a new one, and it will be difficult to maintain the premises upon which the doctor's conclusion in this connection is based. Since cementum assumes so nearly the form of bone the two processes of formation must be very much the same and consequently produced by similar organs. Cementum is claimed—and most properly—to be a sub-periosteal product stimulated into growth by the same causes that effect the formation of sub-periosteal bone. The difference between it and true bone is possibly due to the confined limits in which this tissue is necessarily deposited. Since the periodental membrane, according to Sudduth, has the special superintendence of this deposit, and is simply a continuation of the periosteum, the modification compared to bone can be accounted for easily in the manner described. But what part the enamel organ can possibly have in the production of cementum, either primarily or secondarily, is not easy to see. Moreover, the tissues necessary for the production of enamel are not at all necessary for the production of bone or cementum.

As to the source from which the cords of the permanent teeth are derived authors differ. But the latest investigators almost universally believe that the cords for the twenty anterior permanent teeth are developed from the cords of the twenty corresponding temporary teeth instead of from the epithelium as the essayist thinks, and the cord of the first permanent molar from the epithelium, that of the second molar from the cord of the first, the second in time giving off a cord for the third and last tooth. There are specimens showing deviations from this rule but they seem to be mere irregularities.

In closing my remarks I deem it just to say that since there is no standard in this matter, on account of the extreme difficulties surrounding such minute investigations, all that we can do is to compare the work of the essayist with that done by other late investigators. The training and labor necessary even to follow the steps of the established processes and phenomena in this work are not small. And when one has become so familiar with the microscope and the preparation of specimens that he has perfect mechanical familiarity with his work, then comes the original work which he must undertake. But just here we meet with our greatest difficulty, for we have reached the limit of our appliances and reagents. We can now only let the imagination go on and, reasoning from homologies and analogies, form theories and try to establish them. As improvements may hereafter be made in our means of investigation our theories must stand or fall as they are found to be based upon facts or fiction. And it must be remembered that these improvements are generally brought about by the discoveries and the demands of just such faithful workers as our essayist. To him, therefore, are due the most hearty thanks of all interested in this or in kindred work.

## Figures and Tables

**No. 1. f1:**
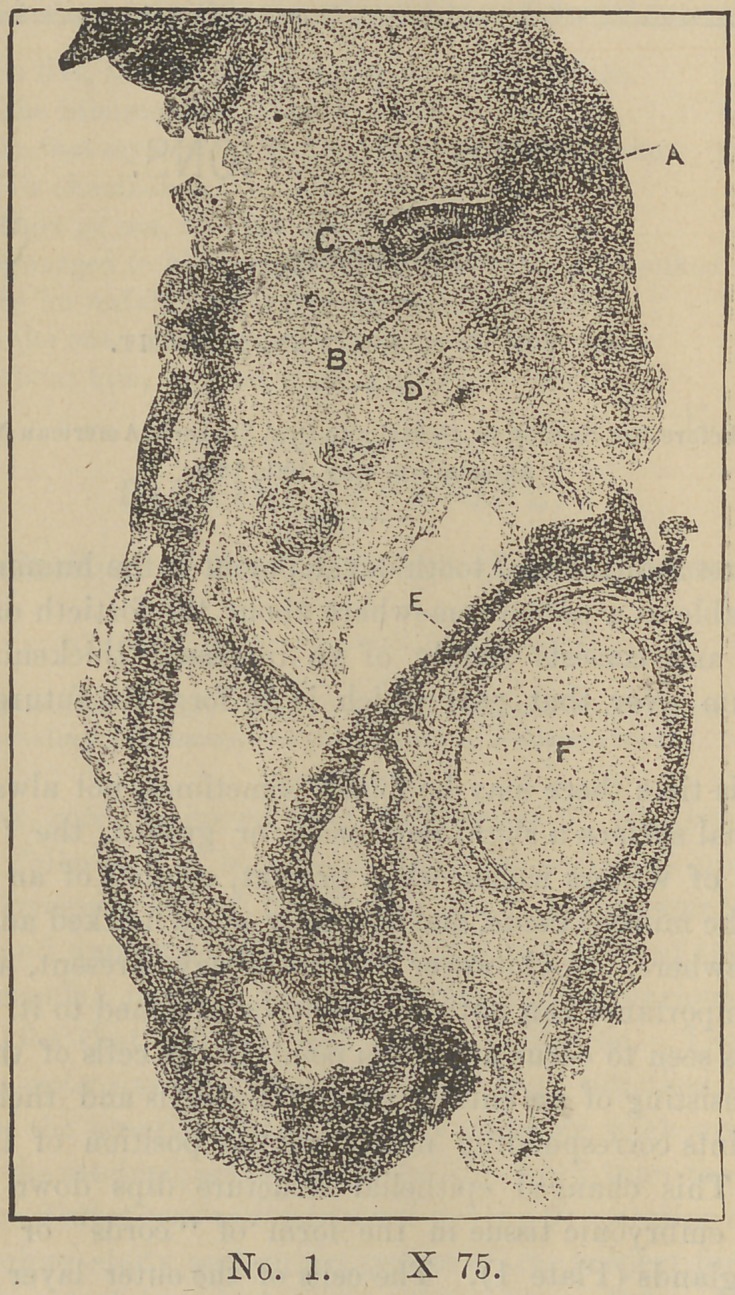


**No. 2. f2:**
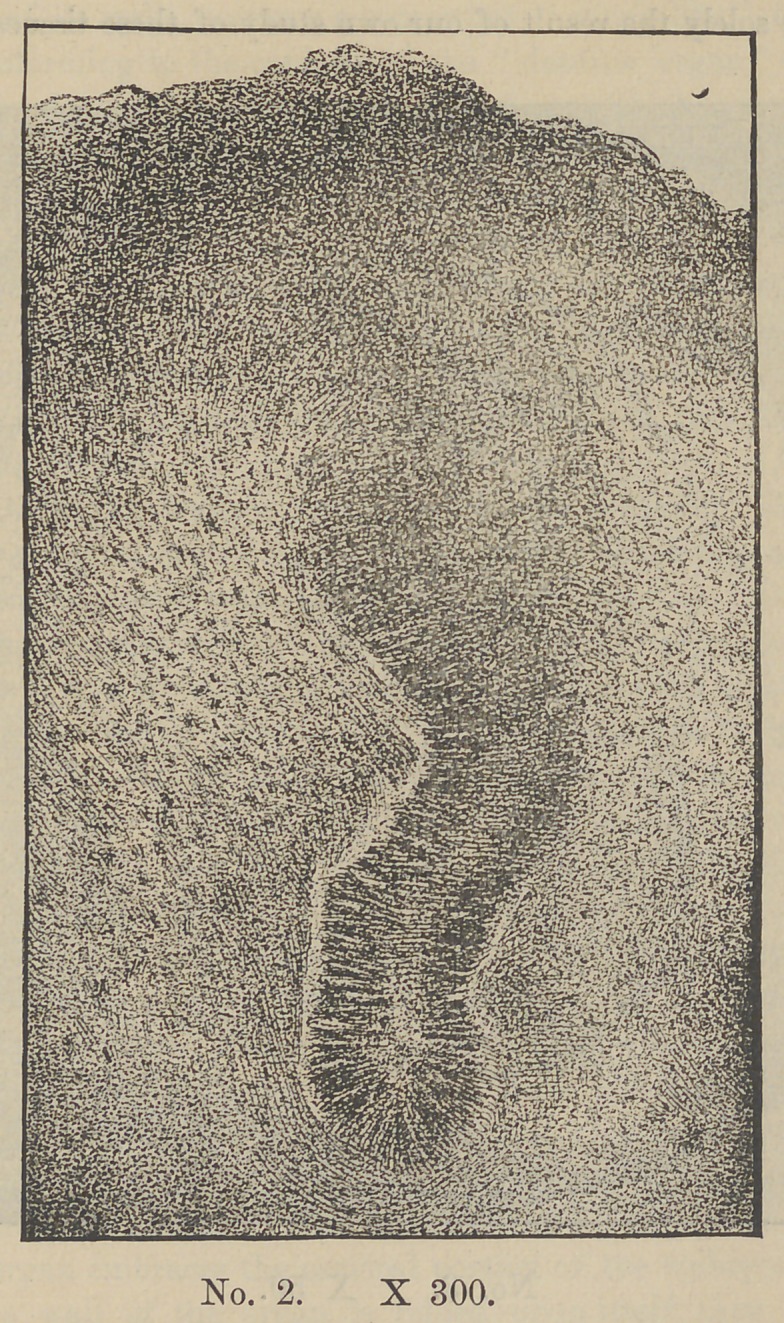


**No. 3. f3:**
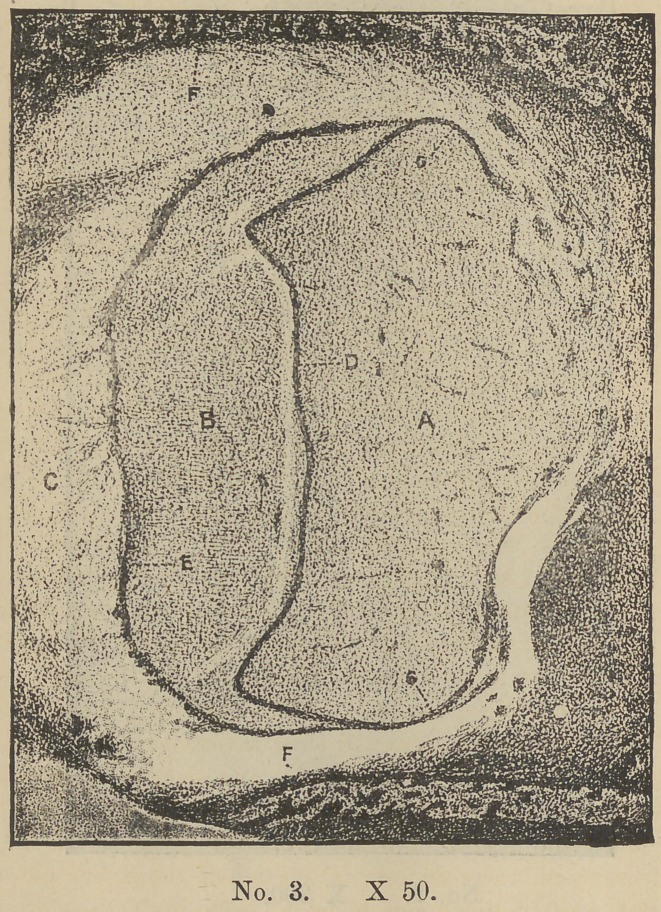


**No. 4. f4:**
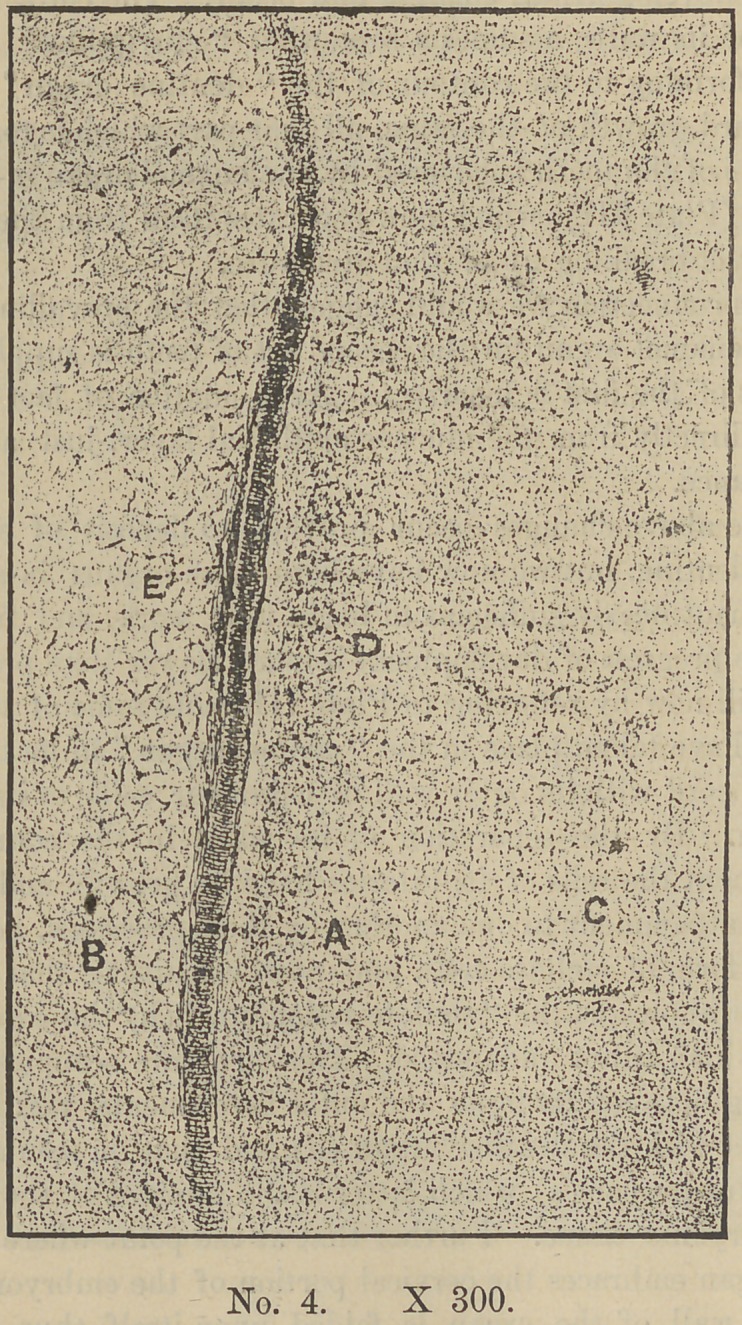


**No. 5. f5:**
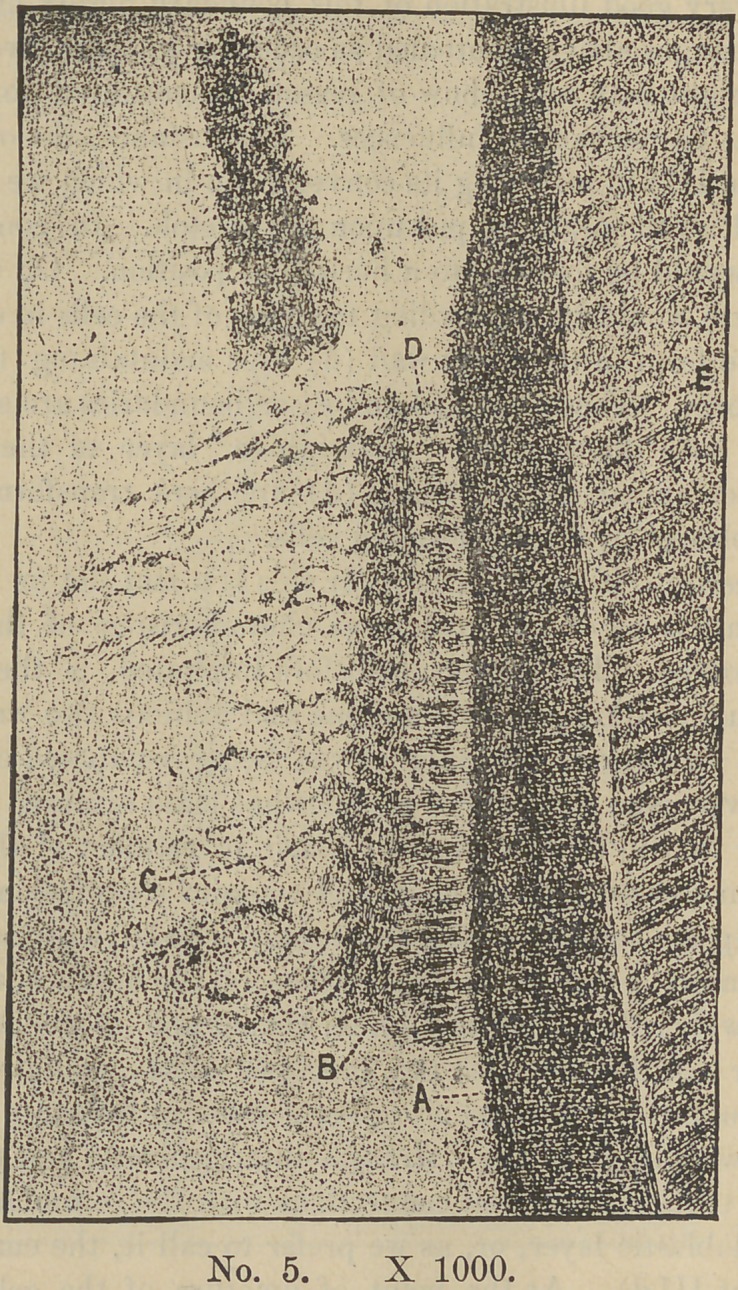


**No. 6. f6:**
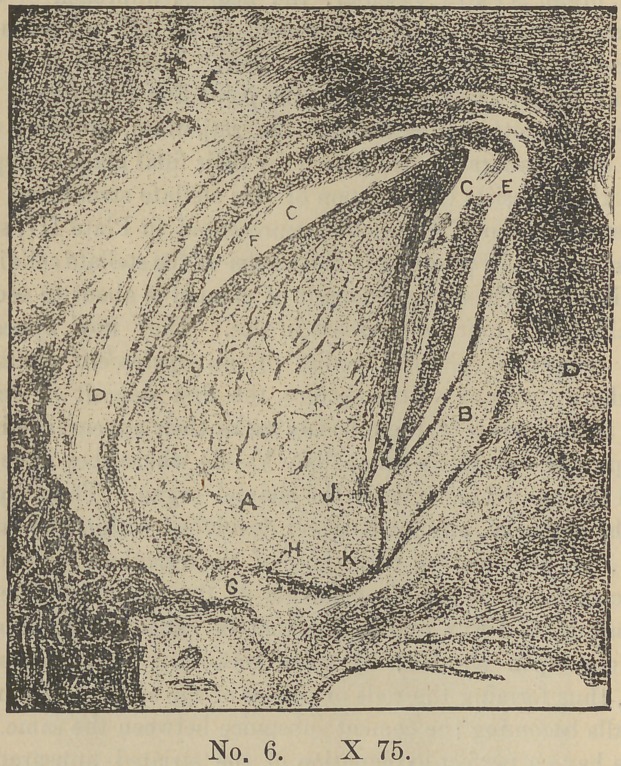


**No. 7. f7:**
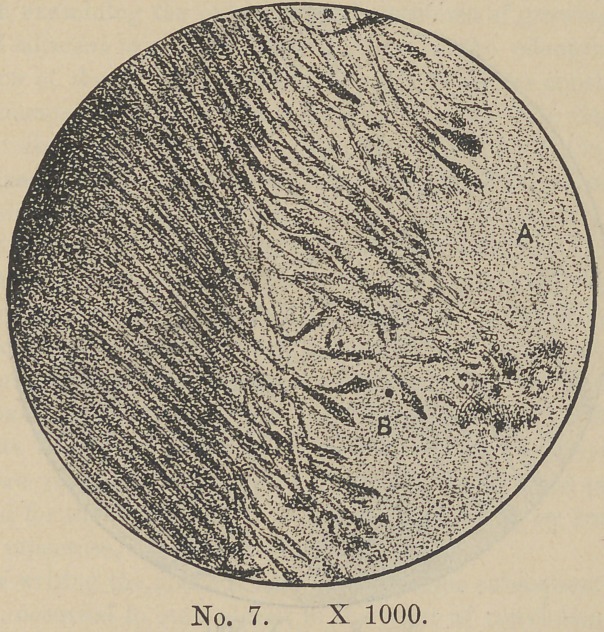


**No. 8. f8:**